# Dyslipidemia Treatment and Lipid Control in US Adults with Diabetes by Sociodemographic and Cardiovascular Risk Groups in the NIH Precision Medicine Initiative *All of Us* Research Program

**DOI:** 10.3390/jcm12041668

**Published:** 2023-02-20

**Authors:** Meleeka Akbarpour, Divya Devineni, Yufan Gong, Nathan D. Wong

**Affiliations:** 1 Heart Disease Prevention Program, Division of Cardiology, University of California, Irvine, CA 92697, USA; akbarpom@hs.uci.edu (M.A.); devineni@hs.uci.edu (D.D.); 2 Department of Epidemiology, University of California, Los Angeles, CA 90095, USA; ivangong@ucla.edu

**Keywords:** dyslipidemia, diabetes mellitus, LDL-cholesterol, triglycerides, statins, treatment

## Abstract

Real-world data on lipid levels and treatment among adults with diabetes mellitus (DM) are relatively limited. We studied lipid levels and treatment status in patients with DM across cardiovascular disease (CVD) risk groups and sociodemographic factors. In the *All of Us* Research Program, we categorized DM as (1) moderate risk (≤1 CVD risk factor), (2) high risk (≥2 CVD risk factors), and (3) DM with atherosclerotic CVD (ASCVD). We examined the use of statin and non-statin therapy as well as LDL-C and triglyceride levels. We studied 81,332 participants with DM, which included 22.3% non-Hispanic Black and 17.2% Hispanic. A total of 31.1% had ≤1 DM risk factor, 30.3% had ≥2 DM risk factors, and 38.6% of participants had DM with ASCVD. Only 18.2% of those with DM and ASCVD were on high-intensity statins. Overall, 5.1% were using ezetimibe and 0.6% PCSK9 inhibitors. Among those with DM and ASCVD, only 21.1% had LDL-C < 70 mg/dL. Overall, 1.9% of participants with triglycerides ≥ 150 mg/dL were on icosapent ethyl. Those with DM and ASCVD were more likely to be on high-intensity statins, ezetimibe, and icosapent ethyl. Guideline-recommended use of high-intensity statins and non-statin therapy among our higher risk DM patients is lacking, with LDL-C inadequately controlled.

## 1. Introduction

Atherosclerotic cardiovascular diseases (ASCVD) are major causes of morbidity and mortality in people with diabetes mellitus (DM) [1]. Dyslipidemia remains a significant ASCVD risk factor in those with DM. In the US Diabetes Collaborative Registry [2], among the 74,393 patients with DM, 48.6% had controlled levels of low-density lipoprotein-cholesterol (LDL-C) but only 62% were on a moderate- or high-intensity statin. Hypertriglyceridemia (HTG) also remains common in patients with DM. Among 1448 U.S. adults aged 20 years and over with diabetes in the US National Health and Nutrition Examination Survey, approximately 40% had triglyceride levels of ≥ 150 mg/dL, regardless of statin use; and even among statin users with LDL-C < 70 mg/dL, one-third had borderline or elevated levels [3]. Moreover, clinical trials have shown that statin therapy, proprotein convertase subtilisin/kexin type 9 (PCSK9) inhibitor use, and fish oil therapy using pure icosapent ethyl all reduce ASCVD risk, including among those with DM [4,5,6].

US and other international guidelines recommend statin therapy for all adults with DM, with high-intensity statins for those at higher risk and icosapent ethyl (pure EPA fish oil) for those at higher risk who have elevated triglycerides [5,7]. US and European guidelines for the management of dyslipidemias now include the use of PCSK9 inhibitors for very high-risk ASCVD patients (with or without DM) who are not adequately controlled for LDL-C on a maximum tolerated dose of statin and ezetimibe [8,9,10].

Data on the extent of dyslipidemia and lipid target attainment, as well as on the use of statin and newer non-statin therapies, are limited among recent real-world cohorts of diverse patient populations. The aim of our study was to examine disparities in lipid control and use of statin and newer lipid therapies according to sociodemographic and ASCVD risk groups in a large cohort of patients with DM representative of the diversity of the United States. Key objectives were to examine differences in (1) LDL-C and triglyceride control by sociodemographic and ASCVD risk groups, and (2) the use of statin, ezetimibe, PCSK9 inhibitor, and icosapent ethyl by sociodemographic and ASCVD risk groups.

## 2. Materials and Methods

### 2.1. All of Us Research Program

The mission of the *All of Us* Research Program is to accelerate health research and medical breakthroughs, enabling individualized prevention, treatment, and care [11]. The *All of Us* Research Program is an ongoing program that aims to invite 1 million adults across the United States. There are currently over 541,000 participants that have been recruited from 590+ sites. Over 50% of these participants represent racial and ethnic minorities, and over 80% of them are underrepresented in biomedical research [11].

This work was performed on data collected using the *All of Us* Researcher Workbench, a cloud-based platform where approved researchers can access and analyze data [11]. The data currently includes surveys, electronic health records (EHR) data, and physical measurements (PM). Participants could choose not to answer specific questions. PM recorded at enrollment include systolic and diastolic blood pressure, height, weight, heart rate, waist and hip measurement, wheelchair use, and current pregnancy status. EHR data was linked for those participants who consented [11]. All participants provided informed consent to participate in the *All of Us* research program. The current analysis utilized de-identified data.

### 2.2. Study Sample

On the researcher workbench, we created a cohort of 81,332 participants aged ≥ 18 years enrolled between 2018 and 2022 with DM based on ≥1 of the following from recorded personal or medical history: DM, DM without complications, type 2 DM, different diseases/conditions due to DM, secondary DM, on insulin treatment or DM medication, HbA1c ≥ 6.5%, fasting glucose ≥ 126 mg/dL, or non-fasting glucose ≥ 200 mg/dL. We excluded participants with Type 1 DM and variables with missing values in our analysis from participants. Ethnicity within our cohort included non-Hispanic White, non-Hispanic Black, Hispanic or Latino, Asian, and other. We categorized our ASCVD risk groups as moderate risk based on ≤1 CVD risk factor, high risk with ≥2 CVD risk factors, and DM with known ASCVD. Risk factors included were age ≥60 years, hypertension (blood pressure ≥ 130/80 mmHg or being on antihypertensive therapy), low-density lipoprotein cholesterol (LDL-C) ≥ 160 mg/dL, cigarette smoking, and high-density lipoprotein cholesterol (HDL-C) < 40 mg/dL for males and <50 mg/dL for females (Table 1). We also analyzed these parameters across health insurance status, education, and income categories.

### 2.3. Definitions and Measurements

We extracted information on each subject on demographics, survey data, cholesterol, LDL-C, and triglyceride levels, as well as use of statins and PCSK9 inhibitor use. ASCVD was defined based on all listed manifestations of coronary artery disease, cerebrovascular disease (excluding hemorrhagic stroke), and peripheral arterial disease. Statin use was defined as a documented prescription (generic or branded) of atorvastatin, cerivastatin, fluvastatin, lovastatin, pitavastatin, pravastatin, rosuvastatin, and/or simvastatin. Statin intensity was categorized into those at high and low/moderate intensities according to US guidelines [12]. Ezetimibe and icosapent ethyl use was also captured, and PCSK9 inhibitors included evolocumab and alirocumab. We additionally obtained survey data on health insurance status, types of health insurance, BMI, education level, cigarette smoking status, and income.

### 2.4. Statistical Analyses

R programming was used for statistical analysis, utilizing the *All of Us* Research Program participants to project estimates to the US population. The Chi-squared test of proportions was used to compare icosapent ethyl and statin use according to risk group, sex, and ethnicity. We examined the percentage of people on low-, moderate-, and high-intensity statin therapy, and at LDL-C levels less than 70 mg/dL, 70–99 mg/dL, and 100 mg/dL or greater. The percentage of people on icosapent ethyl and with triglyceride levels less than 100 mg/dL, 100–149 mg/dL, 150 mg/dL to 199 mg/dL, and 200 mg/dL and greater were also analyzed using the Chi-squared test of proportions. We then performed logistic regressions that assessed the relation of demographic factors to high-statin, ezetimibe, PCSK9 inhibitor, and icosapent ethyl uses. Multiple logistic regressions were used to assess the relation of predetermined sociodemographic factors, risk groups, and individual risk factors, with odds ratios (ORs) and 95% confidence intervals calculated. The *p*-values shown represent the significance levels across the strata of interest (e.g., sex, ethnicity, or DM risk group).

## 3. Results

Our analysis includes 81,332 participants diagnosed with DM based on our inclusion criteria. Overall, 31.1%, 30.3%, and 38.6% were at moderate risk, high risk, or with ASCVD, respectively. Our sample also comprised 22.3% non-Hispanic Black, 17.2% Hispanic or Latino, 52.3% non-Hispanic White, and 1.8% Asian participants, as well as 40.6% males and 59.4% females. Overall, 4.4% did not have health insurance, and 34.1% had a high school education or less (Table 1).

Table 2 shows how the use of different therapies for dyslipidemia varied by risk and demographic groups. Within risk groups, sex, and ethnicity, there were significant differences in the use of statins. Approximately 33.5% of people who have DM and ASCVD were not using any statins. High-intensity statin use also varied among groups, ranging from 5.9% in those at lower risk to 18.2% in those with DM and ASCVD (*p* < 0.05). Furthermore, across all risk groups, use of PCSK9 inhibitors and icosapent ethyl was universally low, being highest at 1.3% and 1.7%, respectively, in those with both DM and ASCVD. Approximately 1.9% of participants with TG levels greater than or equal to 150 mg/dL were on icosapent ethyl. A total of 5.1% of participants were on ezetimibe (*p* < 0.05).

**Figure 1:**
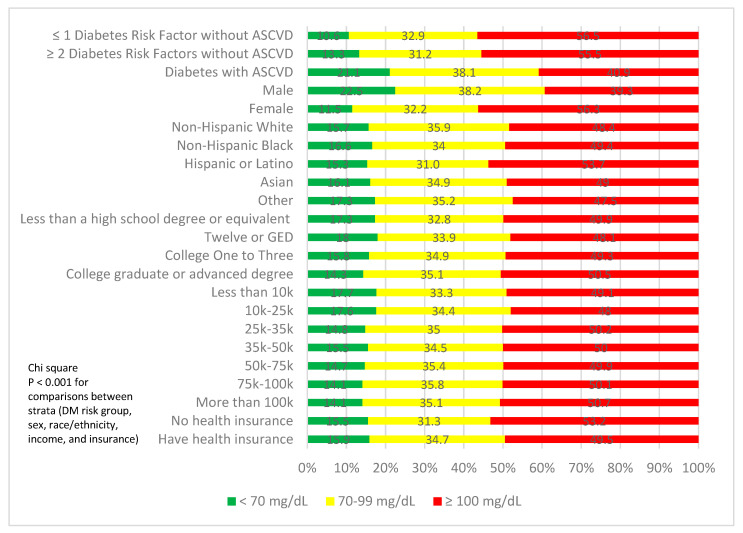
Proportion of subjects at ideal, borderline, or poor LDL-C control by ASCVD risk group, sex, ethnicity, education, income, and health insurance. *p* < 0.001 across risk, sex, ethnicity, education, income, and health insurance status categories.

Overall, 50.6% of our participants had LDL-C levels < 100 mg/dL, although only 16.0% were <70 mg/dL (Table 2). Figure 1 shows the proportion of participants with LDL-C < 70 mg/dL, 70–99 mg/dL, and ≥100 mg/dL according to sociodemographic and ASCVD risk groups. A total of 55.5% of those with ≥ 2 risk factors had LDL-C ≥ 100 mg/dL, whereas 40.9% of those with DM and ASCVD had LDL-C ≥ 100 mg/dL (with only 21.1% having LDL-C < 70 mg/dL). A total of 56.3% of females had LDL-C ≥ 100 compared to 39.3% of males. Of the participants who had health insurance, 49.5% had LDL-C ≥ 100 mg/dL, compared to 53.2% of participants who had no insurance and had LDL-C ≥ 100 mg/dL.

**Figure 2:**
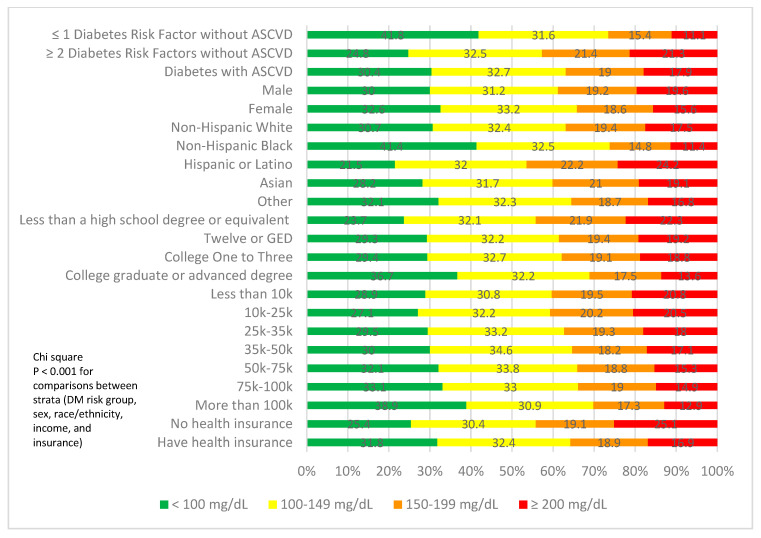
Proportion of subjects at ideal, borderline, or poor triglyceride control by ASCVD risk group, sex, ethnicity, education, income, and health insurance. *p* < 0.001 across risk, sex, ethnicity, education, income, and health insurance status categories.

Overall, 64.4% had triglyceride levels < 150 mg/dL, and only 31.6% had levels < 100 mg/dL (Table 2). Figure 2 shows the proportion of participants with triglyceride levels of <100 mg/dL, 100–149 mg/dL, 150–199 mg/dL, and ≥200 mg/dL by sociodemographic and risk groups. A total of 21.3% of participants with 2 or more risk factors had triglyceride levels ≥ 200 mg/dL, whereas 17.9% of those with DM and ASCVD had triglyceride levels ≥ 200 mg/dL. A total of 15.6% of females had triglyceride levels ≥ 200 mg/dL compared to 19.6% of males. Additionally, 16.9% of participants with health insurance had triglyceride levels ≥ 200 mg/dL, compared to 25.1% of those without health insurance.

Table 3 shows significant differences in the prevalence of high-intensity statin, ezetimibe, PCSK9 inhibitor, and icosapent ethyl use across health insurance, education, and income. For those with health insurance, 27.5% were on high-intensity statins, compared to 23.7% without health insurance. Ezetimibe use was greater in those with health insurance, at 5.3%, compared to 1.4% in those without health insurance. Moreover, 3.1% and 0.5% of participants with less than a high school degree were on ezetimibe and icosapent ethyl, respectively. For those with a college or advanced degree, this was 6.4% and 1.0%, respectively. Ezetimibe use was more common in those with higher versus lower income levels.

Multiple logistic regression (Table 4) showed males to be significantly more likely to be on icosapent ethyl (OR = 2.98 [2.03, 4.48]) and high-intensity statins (OR = 1.73 [1.62, 1.85]) compared to females. Non-Hispanic Black participants were significantly less likely to be on icosapent ethyl (OR = 0.22 [0.12, 0.38]) and ezetimibe (OR = 0.62 [0.54, 0.72]) than non-Hispanic White participants, but were more likely to be on PCSK9 inhibitors and high-intensity statins. High-intensity statin use was significantly more likely in participants with hypertension (OR = 1.13 [1.07, 1.19]), and those with LDL-C ≥ 160 mg/dL (OR = 1.63 [1.43, 1.86). Ezetimibe use was significantly more likely in participants ≥60 years (OR = 1.27 [1.05, 1.54]) and among those with health insurance (OR = 1.52 [1.03, 2.35]). Hispanic or Latino participants were significantly less likely to be taking ezetimibe. Those with DM and ASCVD were significantly more likely to be on a high-intensity statin (OR = 3.66 [3.37, 3.97]) and ezetimibe (OR = 3.12 [2.66, 3.67]) as well as icosapent ethyl (OR = 2.21 [1.44, 3.47]).

## 4. Discussion

We demonstrated continuing gaps in lipid treatment and inadequate control of LDL-C and triglycerides in an important current real-world cohort of US adults with DM. We analyzed these gaps across ASCVD risk groups and key underserved demographic groups of participants within the NIH Precision Medicine Initiative’s *All of Us Study* who have been underrepresented in health research. We found that LDL-C and triglyceride levels remain inadequately controlled, including among people with ASCVD, who despite having the strongest recommendations for treatment, remain suboptimally treated with high-intensity statins, ezetimibe, PCSK9i, and icosapent ethyl. Among participants with both DM and ASCVD, only 21.1% had LDL-C < 70 mg/dL and 36.5% had triglyceride levels ≥ 150 mg/dL, respectively. Additionally, ezetimibe, PCSK9i, and icosapent ethyl, while not widely used, were most prevalent among those with a college degree or higher, and PCSK9i was most used in those with health insurance.

Furthermore, ezetimibe, PCSK9 inhibitor, and icosapent ethyl use were highest among non-Hispanic White populations compared to other minority racial/ethnic groups. These results are concerning because Hispanic or Latino populations and non-Hispanic Black populations had the highest proportions with LDL-C levels ≥ 100 mg/dL, and Hispanic or Latino populations and Asian populations had the highest proportion of uncontrolled triglyceride levels of 150 mg/dL or higher. Others have also shown minority groups are more likely to have high triglyceride levels and low HDL-C dyslipidemia [13]. In the US Diabetes Collaborative Registry [2], we recently showed Black persons to be less likely to be at LDL-C target (42.7%) compared to White persons (49.3%). Moreover, from analysis of electronic health record data from a large healthcare system [14], among those with diabetes, Black persons had a 36% lower likelihood of being prescribed a statin compared to White persons in adjusted analysis. The Reasons for Geographic and Racial Differences in Stroke (REGARDS) study similarly showed underutilization of statins in non-Hispanic Black populations compared to non-Hispanic White populations [15].

Clinical trials have documented the efficacy of statin and ezetimibe therapy as well as PCSK9 inhibitors and icosapent ethyl, including among persons with DM. In 14 randomized statin trials, which included 18,686 people, researchers found that people with DM who were on statins for an average of 4.3 years had a 21% decrease in major vascular events and a 9% decrease in mortality compared to those who were not on statins [4]. Further reduction of LDL-C not satisfactorily achieved by high-intensity statins can be achieved by ezetimibe or PCSK9 inhibitors [4]. In the IMPROVE-IT trial comparing the addition of ezetimibe to statins alone in persons with a recent acute coronary syndrome, in subgroup analyses, those with DM (in addition to the recent acute coronary syndrome) compared to those without DM had a substantially greater reduction in risk of the primary composite cardiovascular endpoint [16]. In the Fourier trial of evolocumab in persons with prior ASCVD, pre-specified subgroup analyses showed that among the 11,031 (40%) patients with DM, there was a similar 17% risk reduction of the primary cardiovascular endpoint compared to the 13% risk reduction in those without DM (interaction term not significant) [5]. Another study found that the rosuvastatin/ezetimibe combination is safe and effective in patients with hypercholesterolemia or dyslipidemia with or without DM and with or without cardiovascular disease [17,18]. The drug combination enabled higher proportions of patients to achieve recommended LDL-C goals than rosuvastatin monotherapy, without additional adverse events [17,18].

However, despite statin use, people with well-controlled LDL still have residual ASCVD risk associated in part with elevated triglycerides that may be lowered by omega-3 fatty acids, such as icosapent ethyl [5] or fibrate therapy. In the REDUCE-IT trial testing the efficacy of icosapent ethyl in persons with prior ASCVD or DM and multiple risk factors with triglycerides of 135–499 mg/dL on statin therapy, those with vs. without DM had a similar risk reduction in the primary endpoint (23% vs. 27%, with the interaction term not significant) [8]. The recently reported RESPECT-EPA trial [19], while of borderline significance for the primary endpoint, did achieve the secondary endpoint, with relative risk reductions due to icosapent ethyl therapy consistent with REDUCE-IT. However, the recently reported PROMINENT trial [20] involving pemafibrate failed to demonstrate any benefit from this therapy in reducing ASCVD risk in persons with DM who had elevated triglycerides and low HDL-C, and instead showed increased LDL-C levels in the treated group.

Recent real-world evidence from population studies in those with DM shows use of lipid-lowering therapy is still limited, and acceptable LDL-C levels are often not achieved. While our recent report from the National Health and Nutrition Examination Survey 2013-2016 did show more than 80% of those with DM were on lipid-lowering therapy, only 57% (among those without ASCVD) had an LDL-C < 100 mg/L and only 26% of those who had both DM and CVD had an LDL-C < 70 mg/dL [21]. Moreover, our recent report from the Diabetes Collaborative Registry showed that 49% of those with DM were at LDL cholesterol targets < 100 mg/dL or < 70 mg/dL if with ASCVD, with two-thirds of these on moderate or high-intensity statins [2]. Our results from the *All of Us* cohort show lower levels of lipid treatment, as well as lower levels at appropriate LDL-C levels, likely due to the greater proportions of underrepresented and/or inadequately insured persons in our cohort.

We have previously demonstrated in US adults with DM that despite statin therapy, triglycerides of ≥150 mg/dL are still present in 40%, and even if LDL-C < 100 mg/dL in those on statin therapy, more than a third of such persons still have triglycerides ≥ 150 mg/dL, warranting the consideration of additional triglyceride reducing therapies [3]. We found icosapent ethyl use to be only 1.9% among participants with triglyceride levels greater than or equal to 150 mg/dL. This low use is consistent with other recent real-world data. A recent study by Derington et al. created cohorts using the National Health and Nutrition Examination Surveys (NHANES) 2009–2014 and the Optum Research Database (ORD) to see how many participants were eligible to receive icosapent ethyl [22]. They estimated 3.6 million US adults to be eligible and observed that the 5-year first event (composite of cardiovascular death, nonfatal myocardial infarction, nonfatal stroke, unstable angina requiring hospitalization, or coronary revascularization) rate without IPE was 19.0% compared to 13.1% with 5 years of IPE treatment, preventing 212,000 events. They also projected that the total 5-year event rate (first and recurrent) could be reduced from 42.5% to 28.9% with 5 years of IPE therapy, preventing around 490,000 events, which would amount to approximately USD 2.6 billion in net annual cost. In addition, because icosapent ethyl was approved for ASCVD risk reduction by the FDA recently in December of 2019, it is not surprising that uptake is low in the current study, especially given the wide range of demographic groups included in the *All of Us* research program.

While our results show those with both DM and ASCVD were most likely to be on high-intensity statins, ezetimibe, and icosapent ethyl compared to people with DM who did not have ASCVD, their use was still suboptimal. High-intensity statins are recommended for those with DM and ASCVD [23,24], with further non-statin therapy indicated for further LDL-C lowering. Only 18.2% of our patients with DM and ASCVD were on high-intensity statins, and only 9.1%, 1.3%, and 1.7% were on ezetimibe, PCSK9 inhibitors, and icosapent ethyl, respectively.

Our study has some strengths and limitations. The participants in this study reflect the diversity of the United States and the data are available in near-real time, which is valuable when trying to understand current lipid treatment and control patterns. While the data are extracted from an on-line platform for analysis, these data are from the NIH Precision Medicine Initiative *All of Us* Research Program that does have standardized methods for data collection regarding surveys and blood measurements. However, like with most research studies, participation is voluntary and thus the sample studied, while large, is not necessarily representative of the US population. Moreover, this is a cross-sectional study and we do not have multiple measures of medication use to assess adherence nor multiple laboratory measures to examine the effects of individual therapies, which would require a clinical trial design. There are also other limitations in using electronic health records (EHR) data, where there may be inconsistencies across study sites in capturing prescription and diagnostic data. Additionally, assuming the absence of a diagnostic code as an absence of disease may lead to information and/or selection bias. Further, it has been demonstrated that one key source of bias in EHRs is “informed presence” bias, where those with more medical encounters are more likely to be diagnosed with various conditions [25,26]. Lastly, as our study population is enriched in underserved and disadvantaged persons, results may differ compared to results from health claims data from insured persons.

## 5. Conclusions

In summary, our cross-sectional analysis demonstrates important disparities in lipid control, as well as in the use of statins, ezetimibe, PCSK9 inhibitors, and icosapent ethyl in US adults with DM across sociodemographic and DM risk groups. Guideline-recommended use of high-intensity statins and ezetimibe among our higher risk DM patients is lacking, with many having inadequately controlled LDL-C levels. Moreover, icosapent ethyl use remains low, even among those with high TG levels. Continued provider and patient education needs to be prioritized—especially among those at highest risk. However, systematic approaches, including the use of EHR and other automated interventions, are needed to address both remaining clinical inertia and significant remaining gaps between evidence-based guidelines and actual care received.

## Figures and Tables

**Table 1:** Demographic characteristics of participants with Type 2 DM.

Variable	Total (*n* = 81,332)
Age (years)	62.0 (±14.1)
Male	31,887 (40.6%)
Female	46,661 (59.4%)
Non-Hispanic White	42,532 (52.3%)
Non-Hispanic Black	18,100 (22.3%)
Hispanic or Latino	13,986 (17.2%)
Asian	1445 (1.8%)
Other Race/Ethnicity	5269 (6.5%)
**Health Insurance**	74,838 (95.6%)
**Income**	
Less than 10 k	11,678 (19.6%)
10–25 k	11,793 (19.8%)
25–35 k	5994 (10.1%)
35–50 k	6262 (10.5%)
50–75 k	7990 (13.4%)
75–100 k	5673 (9.5%)
More than 100 k	10,046 (16.9%)
**Education**	
Less than a high school degree or equivalent	9527 (12.2%)
Twelve or GED	17,147 (21.9%)
Some College	22,394 (28.6%)
College Graduate/Advanced Degree	29,104 (37.2%)
**BMI (kg/m^2^)**	32.5 (±12.2)
**Smoking Status**	
Non-Smoker	43,071 (54.7%)
Former Smoker	23,383 (29.7%)
Current Smoker	12,229 (15.5%)
**Systolic Blood Pressure (mm Hg)**	129.5 (±14.2)
**Diastolic Blood Pressure (mm Hg)**	76.9 (±9.1)
**Triglycerides (mg/dL)**	145.7 (±85.1)
**LDL-C (mg/dL)**	100.9 (±31.3)
**HDL-C (mg/dL)**	50.1 (±15.2)
**Diabetes Risk and ASCVD Status**	
≤1 Diabetes Risk Factors without ASCVD	24,787 (31.1%)
≥2 Diabetes Risk Factors without ASCVD	24,112 (30.3%)
Diabetes with ASCVD	30,682 (38.6%)
**Diabetes Risk Factors**	
Age ≥60 years	50,768 (62.4%)
Hypertension	42,315 (54.9%)
LDL-C ≥160 mg/dL	1835 (3.4%)
Smoking History	12,229 (15.5%)
HDL-C < 50 mg/dL in females	14,861 (18.3%)
HDL-C < 40 mg/dL in males	8641 (10.6%)

Individual categories do not add up to total sample size due to missing data as follows: 2784 persons did not indicate their sex, 3050 persons did not indicate their health insurance status, 21,896 persons did not indicate their income status, 3160 persons did not indicate their education level, 2649 persons did not indicate their smoking status, 1751 persons did not indicate their diabetes risk and/or ASCVD status.

**Table 2:** Lipid treatment and control in US adults with DM according to risk group, sex, and ethnicity.

Proportion (%)	Total (*n* = 81,332)	≤1 DM Risk Factors w/o ASCVD (*n* = 24,780)	≥2 DM Risk Factors w/o ASCVD (*n* = 24,119)	DM with ASCVD (*n* = 30,682)	Female (*n* = 46,661)	Male (*n* = 31,887)	Non-Hispanic White (*n* = 42,532)	Non-Hispanic Black (*n* = 18,100)	Hispanic or Latino (*n* = 13,986)	Asian (*n* = 1445)	Other Race/ Ethnicity (*n* = 5269)
Statin Category											
No statin use	49.8%	68.5%	50.5%	33.5% *	54.2%	43.2% *	46.2%	53.9%	54.5%	51.8%	51.9%
Low intensity	6.6%	4.7%	6.9%	7.9% *	6.6%	6.5% *	7.6%	4.8%	6.0%	5.3%	6.1% *
Moderate intensity	31.8%	20.9%	32.7%	40.4% *	29.4%	35.7% *	35.1%	28.5%	26.8%	32.8%	30.8% *
High intensity	11.8%	5.9%	9.9%	18.2% *	9.8%	14.6% *	11.2%	12.8%	12.6%	10.2%	11.2% *
Ezetimibe Use	5.1%	2.1%	3.2%	9.1% *	4.7%	5.6% *	6.6%	3.0%	2.8%	5.4%	5.8% *
PCSK9 Inhibitor	0.6%	0.1%	0.2%	1.3% *	0.6%	0.7%	0.8%	0.2%	0.3%	---	1.1% *
Icosapent Ethyl Use	1.0%	0.5%	0.8%	1.7% *	0.5%	1.8% *	1.3%	0.3%	0.9%	2.1%	1.4% *
Among those with TG ≥ 150 mg/dL	1.9%	0.1% ^†^	0.5% ^†^	1.1% ^†^	0.9%	2.3%	2.5%	0.3%	0.5%	2.1%	1.1%
LDL-C Category											
<70 mg/dL	16.0%	10.6%	13.3%	21.1% *	11.5%	22.5% *	15.7%	16.6%	15.3%	16.1%	17.3% *
70–99 mg/dL	34.6%	32.9%	31.2%	38.1% *	32.2%	38.2% *	35.9%	34.0%	31.0%	34.9%	35.2% *
≥100 mg/dL	49.5%	56.5%	55.5%	40.9% *	56.3%	39.3% *	48.4%	49.4%	53.7%	49.0%	47.5% *
Triglyceride Category											
<100 mg/dL	31.6%	41.8%	24.8%	30.4% *	32.6%	30.0% *	30.7%	41.4%	21.5%	28.2%	32.1% *
100–149 mg/dL	32.8%	32.1%	33.0%	33.1% *	33.6%	31.7% *	32.9%	32.9%	32.5%	32.0%	32.7% *
≥150 mg/dL	35.6%	26.1%	42.2%	36.5% *	33.7%	38.3% *	36.4%	25.7%	46.0%	39.7%	35.1% *

^†^ *p* value < 0.01, * *p* value < 0.001 across risk, sex, or ethnic groups. Individual categories do not add up to total sample size due to missing data, as follows: 2784 persons did not indicate their sex, and 1751 persons did not indicate their diabetes risk and/or ASCVD status. Percentages not reported are due to cell sizes < 20.

**Table 3:** Prevalence of high-intensity statin, ezetimibe, PCSK9 inhibitor, and icosapent ethyl treatments in adults with DM across health insurance, education, and income.

Proportion (%)	High-Intensity Statin Use	Ezetimibe Use	PCSK9 Inhibitor Use	Icosapent Ethyl Use
Health Insurance (*n* = 74,838)	27.5% *	5.3% *	0.6% *	1.0%
No Health Insurance (*n* = 3469)	23.7% *	1.4%	---	1.1%
Less than a high school degree (*n* = 9527)	31.1% *	3.1% *	---	0.5% *
Twelfth Grade or GED (*n* = 17,147)	28.1% *	4.2% *	0.5% *	0.9% *
College (*n* = 22,394)	26.6% *	4.9% *	0.6%*	1.1% *
College Graduate or Advanced degree (*n* = 29,104)	25.8% *	6.4% *	0.8% *	1.0% *
Income Less than 10 k (*n* = 11,678)	26.9% *	2.8% *	3.0%	0.5% *
10–25 k (*n* = 11,793)	30.8% *	4.5% *	0.2% *	1.0% *
25–35 k (*n* = 5994)	26.8% *	5.2% *	0.7% *	1.1% *
35–50 k (*n* = 6262)	26.7% *	5.7% *	0.5% *	1.6% *
50–75 k (*n* = 7990)	25.6% *	5.8% *	0.7% *	1.3% *
75–100 k (*n* = 5673)	25.0% *	6.8% *	0.8% *	1.4% *
More than 100 k (*n* = 10,046)	24.9% *	7.2% *	0.9% *	1.1% *

* *p* < 0.001 across health insurance, education, or income categories. (Participants may be in one or more medication class.) Percentages not reported are due to cell sizes < 20.

**Table 4:** Multiple logistic regression of indicators for high-intensity statin, ezetimibe, PCSK9 inhibitor, and icosapent ethyl.

Variable	High-Intensity Statin Use Odds Ratio [95% CI]	Ezetimibe Use Odds Ratio [95% CI]	PCSK9 Inhibitor Use Odds Ratio [95% CI]	Icosapent Ethyl Use Odds Ratio [95% CI]
Age (Per Year)	1.02 [1.016, 1.023]	1.02 [1.017, 1.03]	1.02 [0.99, 1.04]	1.00 [0.99, 1.03]
Gender: Male	1.73 [1.62, 1.85]	0.98 [0.87, 1.099]	1.17 [0.84, 1.63]	2.98 [2.03, 4.48]
BMI (Per kg/m^2^)	1.00 [0.999, 1.003]	1.00 [0.998, 1.004]	0.995 [0.992, 1.00]	1.006 [1.003, 1.009]
Age ≥60 years	1.09 [0.99, 1.20]	1.27 [1.05, 1.54]	0.47 [0.27, 0.79]	1.38 [0.85, 2.28]
HTN	1.13 [1.07, 1.19]	0.91 [0.82, 1.00]	1.13 [0.86, 1.47]	0.92 [0.71, 1.18]
LDL-C ≥ 160 mg/dL	1.63 [1.43, 1.86]	3.02 [2.48, 3.66]	0.15 [0.10, 0.23]	0.63 [0.19, 1.50]
Smoking History	1.11 [1.03, 1.19]	0.87 [0.74, 1.02]	1.07 [0.71, 1.69]	0.61 [0.37, 0.96]
HDL-C < 50 mg/dL in females	1.47 [1.37, 1.58]	1.16 [1.02, 1.32]	1.34 [0.93, 1.96]	2.19 [1.41, 3.45]
HDL-C < 40 mg/dL in males	1.27 [1.17, 1.37]	1.05 [0.91, 1.21]	0.99 [0.67, 1.49]	1.92 [1.43, 2.58]
Ethnicity: Non-Hispanic Black	1.30 [1.21, 1.39]	0.62 [0.54, 0.72]	2.49 [1.60, 4.04]	0.22 [0.12, 0.38]
Hispanic or Latino	1.34 [1.24, 1.45]	0.70 [0.59, 0.84]	1.57 [0.98, 2.64]	0.97 [0.64, 1.43]
Asian	0.96 [0.78, 1.17]	0.88 [0.61, 1.24]	4.87 [1.08, 8.59]	2.30 [1.20, 4.01]
Other	1.05 [0.93, 1.19]	1.05 [0.85, 1.28]	0.65 [0.41, 1.07]	1.17 [0.69, 1.88]
Have health insurance	0.79 [0.69, 0.90]	1.52 [1.03, 2.35]	0.22 [0.01, 0.99]	0.60 [0.32, 1.29]
Income: 10–25 k	0.97 [0.89, 1.05]	1.13 [0.94, 1.35]	0.58 [0.33, 0.98]	0.89 [0.54, 1.48]
25–35 k	0.76 [0.69, 0.84]	1.17 [0.95, 1.45]	0.61 [0.32, 1.14]	1.16 [0.66, 2.03]
35–50 k	0.76 [0.69, 0.84]	1.17 [0.95, 1.44]	0.75 [0.39, 1.42]	1.41 [0.84, 2.39]
50–75 k	0.70 [0.64, 0.77]	1.18 [0.97, 1.44]	0.68 [0.37, 1.22]	1.22 [0.74, 2.05]
75–100 k	0.69 [0.62, 0.77]	1.46 [1.19, 1.80]	0.55 [0.29, 1.02]	1.04 [0.60, 1.83]
>100 k	0.71 [0.65, 0.78]	1.53 [1.27, 1.85]	0.53 [0.29, 0.92]	1.15 [0.71, 1.92]
**Diabetes Risk Group**				
Diabetes with ≥2 Risk Factors	1.18 [1.08, 1.30]	1.11 [0.92, 1.34]	0.80 [0.41, 1.51]	1.18 [0.73, 1.96]
Diabetes with ASCVD	3.66 [3.37, 3.97]	3.12 [2.66, 3.67]	0.14 [0.08, 0.24]	2.21 [1.44, 3.47]

Reference Groups: Gender––female; age ≥60 years-age ≤60 years age; HTN-no HTN, LDL-C ≥ 160 mg/dL- LDL-C ≤ 160 mg/dL; smoking history: no smoking history; HDL-C < 50 mg/dL in females; HDL-C > 50 mg/dL in females; HDL-C < 40 mg/dL in males; HDL-C > 40 mg/dL in males; race: non-Hispanic White; health insurance: none; income: < 10 k, DM risk group < 1 DM risk factor.

## Data Availability

All data utilized in this study are available from the *All of Us* Research Program at https://allofus.nih.gov/ (accessed on 15 February 2023).
