# Correction: Akbarpour et al. Dyslipidemia Treatment and Lipid Control in US Adults with Diabetes by Sociodemographic and Cardiovascular Risk Groups in the NIH Precision Medicine Initiative *All of Us* Research Program. *J. Clin. Med.* 2023, *12*, 1668

**DOI:** 10.3390/jcm13041155

**Published:** 2024-02-19

**Authors:** Meleeka Akbarpour, Divya Devineni, Yufan Gong, Nathan D. Wong

**Affiliations:** 1 Heart Disease Prevention Program, Division of Cardiology, University of California, Irvine, CA 92697, USA; akbarpom@hs.uci.edu (M.A.); devineni@hs.uci.edu (D.D.); 2 Department of Epidemiology, University of California, Los Angeles, CA 90095, USA; ivangong@ucla.edu

## 1. Error in Table 2 and Table 3

In the original publication [1], there was a mistake in reporting in Table 2 and Table 3 a few cells with very small percentages, which imply sample sizes of <20 which was an unintentional violation of the *All of Us* policy to do so. The corrected Table 2 and Table 3 appear below, indicating dashes in the cells where this was the case. The authors state that the scientific conclusions are unaffected. This correction was approved by the Academic Editor. The original publication has also been updated.

**Table 2:** Lipid treatment and control in US adults with DM according to risk group, sex, and ethnicity.

Proportion (%)	Total (*n* = 81,332)	≤1 DM Risk Factors w/o ASCVD (*n* = 24,780)	≥2 DM Risk Factors w/o ASCVD (*n* = 24,119)	DM with ASCVD (*n* = 30,682)	Female (*n* = 46,661)	Male (*n* = 31,887)	Non-Hispanic White (*n* = 42,532)	Non-Hispanic Black (*n* = 18,100)	Hispanic or Latino (*n* = 13,986)	Asian (*n* = 1445)	Other Race/ Ethnicity (*n* = 5269)
Statin Category											
No statin use	49.8%	68.5%	50.5%	33.5% *	54.2%	43.2% *	46.2%	53.9%	54.5%	51.8%	51.9%
Low intensity	6.6%	4.7%	6.9%	7.9% *	6.6%	6.5% *	7.6%	4.8%	6.0%	5.3%	6.1% *
Moderate intensity	31.8%	20.9%	32.7%	40.4% *	29.4%	35.7% *	35.1%	28.5%	26.8%	32.8%	30.8% *
High intensity	11.8%	5.9%	9.9%	18.2% *	9.8%	14.6% *	11.2%	12.8%	12.6%	10.2%	11.2% *
Ezetimibe Use	5.1%	2.1%	3.2%	9.1% *	4.7%	5.6% *	6.6%	3.0%	2.8%	5.4%	5.8% *
PCSK9 Inhibitor	0.6%	0.1%	0.2%	1.3% *	0.6%	0.7%	0.8%	0.2%	0.3%	---	1.1% *
Icosapent Ethyl Use	1.0%	0.5%	0.8%	1.7% *	0.5%	1.8% *	1.3%	0.3%	0.9%	2.1%	1.4% *
Among those with TG ≥ 150 mg/dL	1.9%	0.1% ^†^	0.5% ^†^	1.1% ^†^	0.9%	2.3%	2.5%	0.3%	0.5%	2.1%	1.1%
LDL-C Category											
<70 mg/dL	16.0%	10.6%	13.3%	21.1% *	11.5%	22.5% *	15.7%	16.6%	15.3%	16.1%	17.3% *
70–99 mg/dL	34.6%	32.9%	31.2%	38.1% *	32.2%	38.2% *	35.9%	34.0%	31.0%	34.9%	35.2% *
≥100 mg/dL	49.5%	56.5%	55.5%	40.9% *	56.3%	39.3% *	48.4%	49.4%	53.7%	49.0%	47.5% *
Triglyceride Category											
<100 mg/dL	31.6%	41.8%	24.8%	30.4% *	32.6%	30.0% *	30.7%	41.4%	21.5%	28.2%	32.1% *
100–149 mg/dL	32.8%	32.1%	33.0%	33.1% *	33.6%	31.7% *	32.9%	32.9%	32.5%	32.0%	32.7% *
≥150 mg/dL	35.6%	26.1%	42.2%	36.5% *	33.7%	38.3% *	36.4%	25.7%	46.0%	39.7%	35.1% *

^†^ *p* value < 0.01, * *p* value < 0.001 across risk, sex, or ethnic groups. Individual categories do not add up to total sample size due to missing data, as follows: 2784 persons did not indicate their sex, and 1751 persons did not indicate their diabetes risk and/or ASCVD status. Percentages not reported are due to cell sizes < 20.

**Table 3:** Prevalence of high-intensity statin, ezetimibe, PCSK9 inhibitor, and icosapent ethyl treatments in adults with DM across health insurance, education, and income.

Proportion (%)	High-Intensity Statin Use	Ezetimibe Use	PCSK9 Inhibitor Use	Icosapent Ethyl Use
Health Insurance (*n* = 74,838)	27.5% *	5.3% *	0.6% *	1.0%
No Health Insurance (*n* = 3469)	23.7% *	1.4%	---	1.1%
Less than a high school degree (*n* = 9527)	31.1% *	3.1% *	---	0.5% *
Twelfth Grade or GED (*n* = 17,147)	28.1% *	4.2% *	0.5% *	0.9% *
College (*n* = 22,394)	26.6% *	4.9% *	0.6%*	1.1% *
College Graduate or Advanced degree (*n* = 29,104)	25.8% *	6.4% *	0.8% *	1.0% *
Income Less than 10 k (*n* = 11,678)	26.9% *	2.8% *	3.0%	0.5% *
10–25 k (*n* = 11,793)	30.8% *	4.5% *	0.2% *	1.0% *
25–35 k (*n* = 5994)	26.8% *	5.2% *	0.7% *	1.1% *
35–50 k (*n* = 6262)	26.7% *	5.7% *	0.5% *	1.6% *
50–75 k (*n* = 7990)	25.6% *	5.8% *	0.7% *	1.3% *
75–100 k (*n* = 5673)	25.0% *	6.8% *	0.8% *	1.4% *
More than 100 k (*n* = 10,046)	24.9% *	7.2% *	0.9% *	1.1% *

* *p* < 0.001 across health insurance, education, or income categories. (Participants may be in one or more medication class.) Percentages not reported are due to cell sizes < 20.

## 2. Text Correction

In addition, so that we are not reporting results implying sample sizes of <20, we have revised the text in the Results.

Table 3 shows significant differences in the prevalence of high-intensity statin, ezetimibe, PCSK9 inhibitor, and icosapent ethyl use across health insurance, education, and income. For those with health insurance, 27.5% were on high-intensity statins, compared to 23.7% without health insurance. Ezetimibe use was greater in those with health insurance, at 5.3%, compared to 1.4% in those without health insurance. Moreover, 3.1% and 0.5% of participants with less than a high school degree were on ezetimibe and icosapent ethyl, respectively. For those with a college or advanced degree, this was 6.4% and 1.0%, respectively. Ezetimibe use was more common in those with higher versus lower income levels.

The authors state that the scientific conclusions are unaffected. This correction was approved by the Academic Editor. The original publication has also been updated. The authors acknowledge noncompliance with the All of Us Data and Statistics Dissemination Policy (Data and Statistics Dissemination Policy—User Support (researchallofus.org), stipulating that data reflecting actual or inferred sample sizes of <20 not be reported, and apologize for the breach of trust with the All of Us participants.
